# Live lectures and videos do not differ in relation to learning outcomes of dental ergonomics

**DOI:** 10.1002/cre2.300

**Published:** 2020-05-15

**Authors:** Jukka Leinonen, Marja‐Liisa Laitala, Julia Pirttilahti, Leena Niskanen, Paula Pesonen, Vuokko Anttonen

**Affiliations:** ^1^ Department of Clinical Dentistry UiT The Arctic University of Norway Tromso Norway; ^2^ Research Unit of Oral Health Sciences University of Oulu Oulu Finland; ^3^ Medical Research Center University Hospital of Oulu and University of Oulu Oulu Finland; ^4^ Infrastructure for Population Studies, Faculty of Medicine University of Oulu Oulu Finland

**Keywords:** dentistry, lecturing, physical ergonomics, video‐based learning

## Abstract

**Objectives:**

This study aimed to compare the knowledge attained by third‐year dental students in physical ergonomics altering live lectures and videos in teaching. The second aim was to investigate implementation of the theoretical knowledge on ergonomics into practice.

**Material and methods:**

Forty‐five students divided into two groups attended a live lecture (45 min) or viewed videos (45 min). After the first teaching session, the groups changed parts. All students answered a questionnaire with 13 true or false‐questions on ergonomics at baseline and immediately after both teaching sessions. Friedman's test and Wilcoxon signed rank test were used to compare questionnaire scores of the student groups. Additionally, we photographed 17 randomly selected students 3 months after baseline during a simulation workshop on endodontics. We analyzed the photographs for ergonomic postures using a specific 12‐point checklist.

**Results:**

At baseline, no difference in the knowledge between the two groups was discovered, when both scored 72%. After the first teaching session, significant improvement in both groups (*p* < .05) was found; and there was no statistically significant difference in the scores between the groups (88% in the lecture‐first and 82% in the video‐first group). After the second teaching session, the scores were similar in both groups. Overall all improvement in both groups was significant (*p* < .001). The photograph analysis showed half of the postures being in accord with the ergonomic guidelines.

**Conclusions:**

Both live lectures and videos showed similar outcomes in teaching ergonomics. Implementation of the knowledge on ergonomics is insufficient. Videos provide an easy‐to‐organize alternative to live lectures in teaching dental ergonomics. New means are needed to have dental students implement their knowledge on ergonomics into practice.

## 1 INTRODUCTION

Physical ergonomics is a field of science that comprises designing equipment, devices, and processes that fit the human body (International Ergonomics Association, 2019). The goal of ergonomics is to establish working environment that prevents health problems and consequently improves human productivity (International Ergonomics Association, 2019). Ergonomics has three domains for example, physical, cognitive, and organizational ergonomics. This article is about physical ergonomics and from here on, the word “ergonomics” replaces “physical ergonomics.” In dentistry, physical ergonomics aims to reduce physical stress and occupational diseases as well as to improve productivity (Gupta, Bhat, Mohammed, Bansal, & Gupta, 2014).

Dentists' and dental students' knowledge on ergonomics is scarce, and its implementation far from satisfactory (Garbin, Garbin, Diniz, & Yarid, 2011; Siddiqui, Wali, Khan, Khan, & Zafar, 2016). Poor ergonomics is associated with high prevalence of work‐related musculoskeletal disorders among dentists (Alexopoulos, Stathi, & Charizani, 2004; Botta, Presoto, Wajngarten, Campos, & Garcia, 2018), consequently leading to personal suffering and loss of effective work‐hours. Already during the simulation laboratory teaching two out of three dental students present working postures that predispose to musculoskeletal disorders (Corrocher, Presoto, Campos, & Garcia, 2014). Surprisingly, these nonergonomic working postures are not associated with gender, type of dental procedures or site of the mouth treated (Corrocher et al., 2014). Instead, the musculoskeletal pain reported by dental students is associated with low knowledge on ergonomics, torsion, or cervical flexions to improve field of vision, suboptimal settings in the dentist's chair, and excessive curvature of the vertebrae while working (Diaz‐Caballero, Gómez‐Palencia, & Díaz‐Cárdenas, 2010).

An interesting means to increase the knowledge on ergonomics is learning through videos, especially as the online videos are within the reach of most modern‐day dental students and dentists (Schulz et al., 2013). The aims of this study were to compare learning outcomes of live lectures and videos in ergonomics in third year dental students as well as to investigate implication of the knowledge on ergonomics to practice. Our hypothesis was that learning outcomes are better using live lectures than by using videos.

## 2 MATERIALS AND METHODS

### 2.1 Study population

**FIGURE 1:**
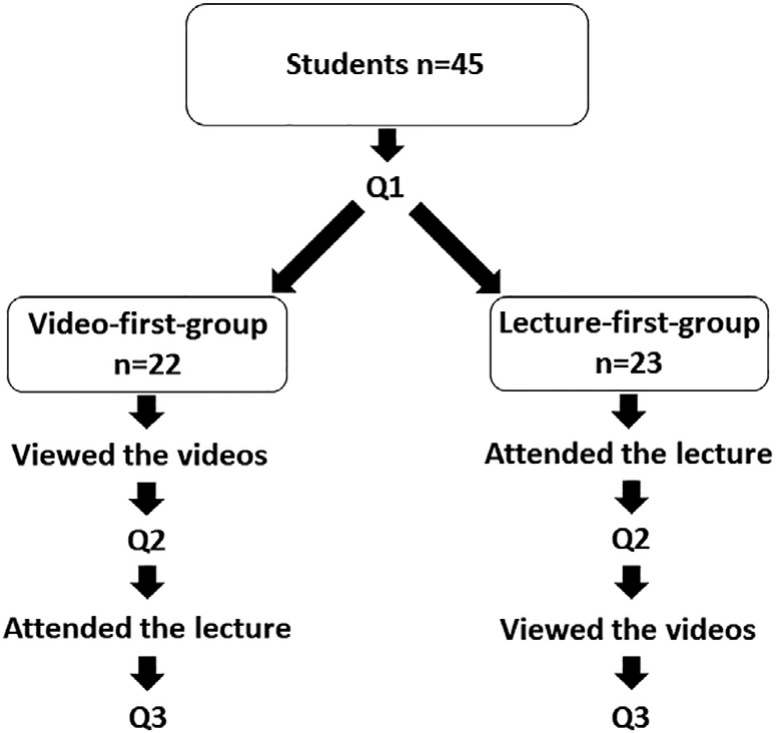
A flowchart representing the course of events in the study (Q = questionnaire)

The study population comprised an entire class of 45 dental students attending their third year of dentistry studies in the University of Oulu, Finland (Figure 1). This is the first time in the curriculum when they are introduced the topic of dental ergonomics. In this survey, the cross‐over randomized controlled trial method was used. We divided the students to two groups based on their previously set study groups. The video‐first‐group comprised 22 dental students and the lecture‐first‐group comprised 23 dental students.

### 2.2 Teaching sessions

Teaching took place simultaneously for both groups (2 x 45 min). After the first teaching session, the groups changed parts. Both groups answered the same questionnaire at baseline as well as immediately after both teaching sessions. Figure 1 illustrates the protocol. One of the authors (L.N.) organized all teaching sessions. The live lecture was given by a physiotherapist specialized in ergonomics through formal lecturing with demonstrations. The video viewing session comprised sections of a video produced by the Finnish Dental Association (2008) on ergonomics to dentists, as well as video clips on dental ergonomics from YouTube. The videos included recordings of patient care simulations plus podcast‐like sections with PowerPoint slides and voiceover. The teacher (L.N.) presented the videos on silver screens but gave no verbal teaching during the session. The contents of the sessions were not identical, but they both covered same topics and gave basic knowledge on dental ergonomics, which enabled the students answer the questions after the session.

### 2.3 The questionnaire

The questionnaire had 14 true or false‐questions that assessed the students' theoretical knowledge on ergonomics. The question on the role of deep abdominal muscles was excluded from the analyses because the students misunderstood it (Appendix A).

### 2.4 Examples of implication of ergonomics teaching

We photographed 17 randomly selected students during their simulation workshop on endodontics 3 months after the ergonomics teaching sessions. The authors J.P. and L.N. analyzed the ergonomics of the students by using a specific 12‐point checklist (Appendix B).

### 2.5 Statistics

The distributions of total sums of correct answers within all questionnaires between the groups were analyzed using Pearson's Chi‐Square test. Friedman's test and Wilcoxon signed rank test were used to compare the total sums of correct answers between the three questionnaires by groups. The changes of total sums were illustrated with line diagrams and the distribution of correct ergonomic components in work posture is illustrated with bar diagram. *p* Values below .05 were considered statistically significant. All analyses were conducted using SPSS software (version 24.0, IBM Corp., Chicago, IL).

### 2.6 Ethical considerations

According to Finnish legislations, there is no need for written consent for cross‐over surveys like the present one containing no personal identifications (https://www.finlex.fi/en/laki/kaannokset/1999/en19990488_20100794.pdf). All participants volunteered to participate in the study. No informed consent was requested, but the participants gave their consent by responding to the questionnaires. The students were informed orally that answering the questionnaires had no influence on their studies or grades—all analyzes were carried out without identifications.

## 3 RESULTS

**FIGURE 2:**
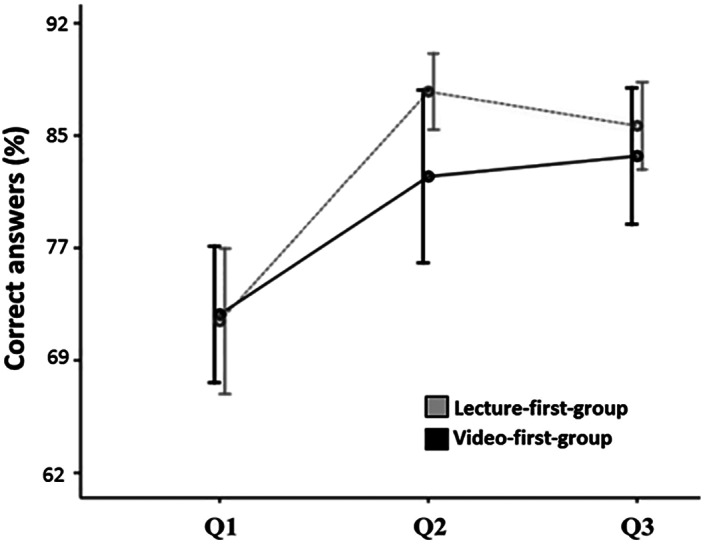
The changes in total sum scores of correct answers in questionnaires 1, 2, and 3 in lecture‐ and video‐first‐groups (Q = questionnaire)

At baseline (Questionnaire 1), the third‐year dental students had good basic knowledge on ergonomics with no difference between the video‐first‐group and lecture‐first‐groups (both scored 72%; Figure 2). After the first teaching session in both groups, the improvement in knowledge was significant (*p* < .05). However, there was some difference in the scores between the groups: the lecture‐first‐group had more correct answers (88%) than the video‐first‐group (82%; *p* = .053). After the second teaching session, the total increase in scores was significant regardless the order of teaching methods (*p* < .001) and the scores in both groups were close to each other (84 and 83%, respectively; Figure 2).

At baseline, the proportion of correct answers was the highest on the question concerning the resting position of the upper arm and the lowest on the question concerning the correct position of the haunch.

**FIGURE 3:**
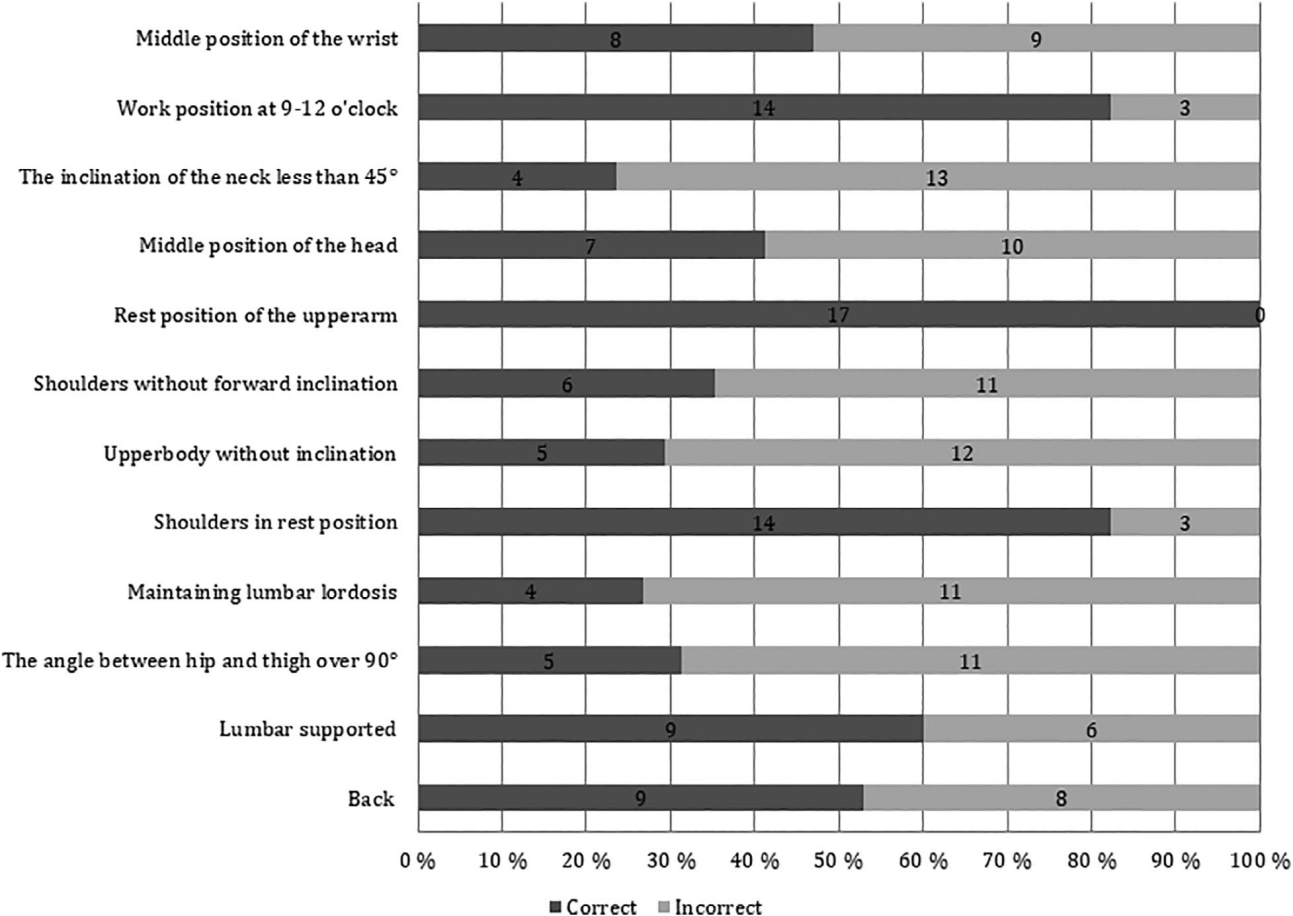
Distribution of correct ergonomic components in work posture analyzed from photographs of 17 dental students during their simulation clinic workshop in endodontics 3 months after teaching dental ergonomics

The biggest improvement occurred in both groups concerning knowledge of working distance from the object and cervical vertebrae bending forward (neck inclination). The students in the lecture‐first‐group had great improvement also concerning positioning of the suction device and foot pedal whereas the video‐first‐group improved knowledge on the question concerning the correct position of the haunch. The analysis of the photographs taken 3 months after baseline showed that the proportion of correct ergonomic positions was 51%. Only 18% of the students had only one or no incorrect components in their working position. The students succeeded best in maintaining their working position “at 9 to 12 o'clock” in relation to the patient and resting position of shoulders and upper arms. The students succeeded least in the inclination of the neck, lumbar lordosis, inclination of the upper body and angle between hip and thigh (Figure 3).

## 4 DISCUSSION

The main outcome of this study was that third‐year dental students with the same background knowledge learnt basic theory on dental ergonomics equally well through live lectures and videos regardless in which order they received information on the topic, or whether they had live lectures or saw videos first. Those who had live lectures first had somewhat better knowledge after the first teaching session than the video first group, but the differences between the groups disappeared after the second session. Unfortunately, implementing theory into practice was poor. Poor implementation of ergonomics observed here after the intervention is a major risk factor for work‐related musculoskeletal disorders, and should be a matter of concern for dental educators.

The average questionnaire score increased after the first teaching session in both groups but more so in the lecture‐first than in the video‐first‐group. Although the difference was not statistically significant, the finding is in accord with earlier studies reporting a small and in most cases nonsignificant difference in learning scores between live lectures and videos (Ramlogan & Raman, 2014; Schönwetter, Gareau‐Wilson, Cunha, & Mello, 2016; Schreiber, Fukuta, & Gordon, 2010; Vaccani, Javidnia, & Humphrey‐Murto, 2016). The students scored nearly three quarters correct answers in the questionnaire even at baseline before any teaching, maybe because the questions were practical, and the students could come up with many correct answers using common sense or simply by trying out different body positions. For research groups planning a similar study, a more challenging questionnaire with preferably multiple‐choice questions could be beneficial in distinguishing a difference between different teaching methods. In addition, if we had shared the videos (as they are shared in practice) so that the students could have used them also in private and on the manner best suited for their individual needs, the questionnaire scores could have improved even more.

On some questions, the average score increased distinctly more after the live lecture and on other questions after the videos. Although both the videos and live lectures gave answers for the questionnaire, the teaching sessions were not fully identical. Different styles of delivering information are optimal for videos and live lectures and therefore comparing outcomes by a recorded lecture to a live lecture is challenging per se. One option could be to produce fully identical video and live lectures sessions and then compare the learning outcomes, which was not done here. The differences in student scoring may result from either presentation being clearer on their expression or the students being more alert by a captivating performance. After all, the lecturer is a major factor in effecting both lecture attendance and learning (Gupta & Saks, 2013; Hattie, 2015).

It may be concluded that the videos in this study were far less captivating in their narrative nature than the live lectures as shown by the outcome after the first session. The questionnaire scores were almost the same in the end of both teaching sessions. This highlights the weight of the first impression in settling an opinion after which the students reached their plateau point of the learning curve during the first 45‐min teaching session. Teaching took place simultaneously for both groups (2 x 45 min). After the first teaching session, the groups changed parts. Both groups answered the same questionnaire at baseline as well as immediately after both teaching sessions.

Strength of the study is recording of ergonomics implication from theory into practice, even if all students were not included in this part of the study. This can be considered to cause bias in the outcome. Unfortunately, a vast majority of the photographs showed poor ergonomics. The students seem to have acted as reported earlier (Garcia, Gottardello, Wajngarten, Presoto, & Campos, 2017) that is, they chose the poor working position despite knowing the correct working position. In some subcategories, the working position was poor despite excellent knowledge whereas on others the outcome was poor in both the questionnaire and practice. Then again, the student excelled in both knowledge and implication of resting position of the upper arm.

The study population was not randomized, but rather a convenience sample. However, the equal scores at baseline show that as for this topic this did not cause bias in the results. Ethical clearance is not necessary in surveys according to Finnish legislation when no identification details are collected, which was the case here. Again, the study, and eventually also the individuals would have benefitted if further analyses allowing investigation of dependent/related variables had been possible.

The retention rate of traditional lectures is low, and lower than for interactive methods (Subramanian, Timberlake, Mittakanti, Lara, & Brandt, 2012). Not surprisingly, 95% of students are unsatisfied with the traditional lecturing as a teaching method, and would prefer activating accompaniments, foremost audio‐visual aids (Jain, Bansal, Singh, & Kumar, 2014). It must be kept in mind that students often have a distorted image of what is the best method for them to learn and remember (Kornell & Bjork, 2007). On some occasions, the students find the videos better than live lectures but in most cases, they prefer to have the videos integrated into the lecture (Hurst, 2016; Ramlogan & Raman, 2014). The students report that videos help them especially in learning clinical manual skills and when learning operations students prefer videos to live demonstrations (Ramlogan & Raman, 2014; Smith, Rafeek, Marchan, & Paryag, 2012). A video demonstration is no doubt superior to words and pictures in demonstrating a clinical procedure.

Surprisingly, the availability of lecture recordings has no apparent effect on the live lecture attendance (Pilarski, Alan Johnstone, Pettepher, & Osheroff, 2008). Instead, the students use recorded lectures if they have had to miss a lecture, to clarify/review the lecture material, to enable the use of individual learning methods, to view the lectures at the most suitable time of day or to use their individual optimal pacing in learning (Topale, 2016). Therefore, the hard‐to‐test ingredients emphasized in live lectures and other classroom teaching, for example, critical thinking will be transmitted in the same extent even if there is video learning material available online.

## 5 CONCLUSION

Viewing videos and attending live lectures resulted in good and similar learning outcomes in learning dental ergonomics by third‐year dental students, just starting their clinical career. Therefore, teaching ergonomics using videos is an option and an alternative to live lectures. Despite the good theoretical learning outcome, only one in five of the students excelled on the implementation of ergonomics into practice. New ways must be found and tested to promote better ergonomics among dental students and dentists.

## CONFLICT OF INTEREST

The authors declare no potential conflicts of interest with respect to the authorship and/or publication of this article.

## APPENDIX A 1

### Web‐based questionnaire (true or false questions)

1. The basis for ergonomic working is correct sitting posture: the angle between the torso and legs should be less than 90°. The height of the dentist's seat affects this angle. True.
2. Declination of the seat should be adjusted to the angle of 5° to 15°. Legs should be firmly on the ground and lower back should be supported by the seat. True.
3. Maintaining lumbar lordosis is essential: support to the lower back and the angle of about 90° between torso and the legs makes this easier to achieve. False.
4. The patient chair should be adjusted so that when treating a patient there would be no need for the dentist to raise his upper arms forwards. Rotating shoulders for 30° forwards causes excess load in vain to both shoulder joints and muscles of the shoulders. True.
5. Forward inclination of vertebra must not exceed 50°. False.
6. One can adjust field of vision only by changing the height of the chair. False.
7. A good working distance is approximately 55–65 cm. False.
8. Right positioning of the instrument tray decreases workload. One can avoid raising upper arms by placing the instrument tray near the ear of the patient so that the instruments would be close to the hand. True.
9. The best place for the suction is at 12–13 o'clock? The foot pedal lays where it is easily available without having have to reach out, that is, so that the balanced working position is not threatened. True.
10. Looking at monitors brings alteration to dentist's daily workload because it causes no strain on neck, shoulders or arms. False.
11. Having frequent cessations reduces harmful strain caused by static working positions. Optimal benefits are achieved by the maximum 1 min “micro breaks” and taking them frequently. True.
12. Breaks are intended to improve circulation in muscles and maintain normal flexibility in joints. It is enough to sit down statically during breaks. False.
13. Stretching ones muscles should be focused on muscles in the neck, shoulders, chest, and back of thigh as well as gluteal muscles, lower back and haunch reducers. True

## APPENDIX B 1

### Evaluation checklist for physical ergonomics of a dentist (yes/no)

Student ID__________________________

Evaluator___________________________

1. Back
  Traditional dentist chair: sitting with straight back; lower back is supported by the chair.
  Saddle chair: correct inclination of thighs; lumbar lordosis.
2. Shoulder is in resting position: (right and left shoulder assessed separately).
3. Upper torso is in correct posture (i.e., not bending, leaning nor twisting).
4. Upper arm close to the sides (right and left arm assessed separately).
5. Head position is appropriate (i.e., not bending, leaning nor twisting overtly).
6. Height of the chair is adjusted correctly.
7. Working position is somewhere between “clock positions” 9 and 12.
8. Wrist is at “middle position” (i.e., not extended or bent).
